# Visual Neuroplasticity: Modulating Cortical Excitability with Flickering Light Stimulation

**DOI:** 10.3390/jimaging11070237

**Published:** 2025-07-14

**Authors:** Francisco J. Ávila

**Affiliations:** Departamento de Física Aplicada, Facultad de Ciencias, Universidad de Zaragoza, 50009 Zaragoza, Spain; avila@unizar.es

**Keywords:** cortical excitability, neuroplasticity, visual stimulation, EEG, critical flicker fusion, Arduino, magnocellular pathway, parvocellular suppression

## Abstract

The balance between cortical excitation and inhibition (E/I balance) in the cerebral cortex is critical for cognitive processing and neuroplasticity. Modulation of this balance has been linked to a wide range of neuropsychiatric and neurodegenerative disorders. The human visual system has well-differentiated magnocellular (M) and parvocellular (P) pathways, which provide a useful model to study cortical excitability using non-invasive visual flicker stimulation. We present an Arduino-driven non-image forming system to deliver controlled flickering light stimuli at different frequencies and wavelengths. By triggering the critical flicker fusion (CFF) frequency, we attempt to modulate the M-pathway activity and attenuate P-pathway responses, in parallel with induced optical scattering. EEG recordings were used to monitor cortical excitability and oscillatory dynamics during visual stimulation. Visual stimulation in the CFF, combined with induced optical scattering, selectively enhanced magnocellular activity and suppressed parvocellular input. EEG analysis showed a modulation of cortical oscillations, especially in the high frequency beta and gamma range. Our results support the hypothesis that visual flicker in the CFF, in addition to spatial degradation, initiates detectable neuroplasticity and regulates cortical excitation and inhibition. These findings suggest new avenues for therapeutic manipulation through visual pathways in diseases such as Alzheimer’s disease, epilepsy, severe depression, and schizophrenia.

## 1. Introduction

### 1.1. Cortical Excitablity

Cortical excitability can be understood as the amplitude of the neural response at the cortex to a sensory stimulation [1]. The balance between excitation and inhibition in the activity of the cortex, also known as E/I balance [2], plays a fundamental role in neuroplasticity, learning, memory and cognitive functions [3]. Neural E/I imbalance is a common feature of many neurological and neuropsychiatric disorders [4]. An increased E/I ratio has been assumed to be the pathophysiological mechanism in autism spectrum disorders [5,6].

The E/I balance mechanism is crucial for maintaining optimal neural network communication and cognitive performance [7]; therefore, E/I imbalance can lead to neural alterations and cognitive impairment [8] and has been related to epilepsy [9], neurodegenerative pathologies such as Alzheimer’s [10] and Parkinson’s disease, multiple sclerosis [11], and psychiatric disorders, including severe depression [12], stress-induced states [13], and schizophrenia [14].

### 1.2. Magnocellular and Parvocellular Visual Pathways

The human visual system has two main, well-characterized neural channels for processing spatial and temporal information, termed parvocellular (P) and magnocellular (M) pathways, respectively [15].

The M-pathway is responsible for motion detection, luminance contrast (achromatic sensitivity), low spatial resolution, and fast temporal changes [16]. In contrast, the P-pathway processes high-spatial resolution, color discrimination, and low temporal changes [17]. Visual P- and M-pathways process the information in a parallel and antagonistic interaction [18]; therefore, the visual manipulation of their relative activation could induce measurable changes in cortical excitability and inhibition.

Visual stimulation is a promising tool for non-invasive neuromodulation, since the human visual system has well-characterized neural pathways that process spatial and temporal information [19]. Understanding how controlled visual stimulation impacts neural circuits could lead to the development of novel therapeutic approaches to treatments for conditions such as major depressive disorder [20], epilepsy [21], Alzheimer’s disease [22], or schizophrenia [23].

### 1.3. Flicker Visual Stimulation

The critical flicker fusion frequency (CFF) is a well-established measure of the temporal resolution of the visual system. It represents the highest frequency at which a flickering light source is perceived as a steady stimulus [24]. The CFF threshold is influenced by retinal eccentricity, stimulus contrast, and neural adaptation [25]. At high flicker frequency, the magnocellular system reaches its physiological threshold, requiring increased neural processing to maintain temporal resolution [26]; that is, the magnocellular–dorsal system is isolated beyond the critical frequency [27].

Neurons in the visual cortex respond to a flicker stimulus at the same frequency of the flickering light itself; those so-called resonant frequencies suggest the presence of neural oscillators that are particularly responsive to specific stimulation frequencies [28].

High-frequency visual stimulation has been shown to enhance neural responses in the primary visual cortex (V1), as measured by increased peak-to-peak amplitudes in visual evoked potentials (VEPs) [29]. Additionally, Western blot assays revealed that this kind of stimulation significantly increases the membrane expression of AMPA and NMDA receptors in V1 [29].

Flickering polychromatic stimuli can entrain neural oscillations, especially in the gamma band (~40 Hz), related to sensory and cognitive processing, which is often altered in Alzheimer’s disease [30,31]. Sahin and Figueiro [32] showed that flickering red light can enhance coherent gamma oscillation, observing a significant increase in 40 Hz power. More recently, acousto-optic [33] and multisensory [34] gamma stimulation have also been explored as non-invasive methods to promote synaptic plasticity, myelination, and reduce neuroinflammation in neurodegenerative conditions.

### 1.4. High Frequency Oscillations and Brain Disorders

The interaction between inhibitory and excitatory neural mechanisms, mainly controlled by the GABAergic and glutamatergic systems, is responsible for cortical excitability [35]. In this sense, high-frequency visual stimulation can modulate both long-term potentiation (LTP) and long-term depression (LTD), which are two mechanisms of synaptic plasticity [29]. Therefore, such stimulation can influence the activity of the thalamocortical pathway, an important circuit controlling attentional and sensory filtering. The thalamus not only acts as a relay station for sensory information but also modulates the patterns of cortical interconnections for higher-order functions, such as attention, in conjunction with the prefrontal cortex [36].

Neural synchronization of oscillatory waves is involved in a wide variety of cognitive functions and perception. However, neural desynchronization in the high-frequency gamma band is associated with brain disorders such as Alzheimer’s disease, autism, epilepsy, Parkinson’s disease, and schizophrenia [37].

Furthermore, excitation of beta waves can generate stress, anxiety, and panic attacks, while their inhibition can lead to depression and poor cognitive performance [38]. Therefore, neural signatures of high-frequency bands can serve as biomarkers of mental health.

### 1.5. Hypothesis

Although the CFF represents the temporal processing limit of the visual system, the introduction of optical scattering can selectively degrade spatial resolution by affecting the parvocellular system responsible for processing fine detail. Scattering occurs when light diffusely spreads across optical irregularities, reducing contrast sensitivity and spatial resolution. This phenomenon has been associated with increased cognitive load and altered neural activation patterns in cortical areas responsible for discriminating fine details.

Sawatari and Callaway [39] found that neurons in the primary visual cortex (V1), particularly those in layer 4B, receive strong input from both magnocellular and parvocellular pathways. This anatomophysiological convergence provides a potential substrate for cortical reorganization if one of these pathways is compromised.

We hypothesize that the induction of optical scattering (which inhibits the P-pathway), when the visual stimulus is presented at the critical fusion frequency, may create a competitive interaction that could lead to cortical reorganization and modulate cortical excitability, where compensatory mechanisms attempt to balance spatial and temporal processing demands.

### 1.6. Objective

Cortical excitability in the somatosensory and visual cortex plays a fundamental role in learning and neural plasticity [3]. Furthermore, as mentioned above, neural modulation of high-frequency bands may contribute to the treatment of brain disorders.

With these premises, the objective of this study is to investigate how Arduino-driven visual flicker stimulation, tuned near the CFF and combined with controlled optical scattering effects, modulates cortical excitability measured from real-time EEG recordings. Our goal is to quantify changes in neural oscillations and provide a new approach to measure the excitation/inhibition (E/I) ratio. This approach provides a novel framework for visual neuromodulation research and could support the development of new interventions for neuropsychiatric and cognitive disorders.

## 2. Materials and Methods

### 2.1. Subjects

A cohort of 19 healthy European Caucasian subjects (age range: 44 ± 3 years old) was selected for the study. None of them had a history of neurological disorders or visual impairment. None of them were taking any medication or drugs that could alter cortical activity at the time of the measurements.

The study was conducted in accordance with the Declaration of Helsinki. All subjects were informed and signed an informed consent form, approved by the Ethics Committee of the Health Sciences Institute of Aragon, Spain. (protocol code: C.P.-C.I. PI20/377, date of approval: 14 July 2020).

### 2.2. EEG Device

**Figure 1:**
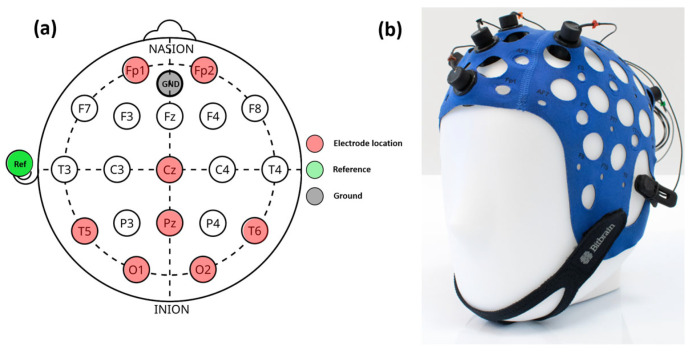
EEG cap configuration (**a**): the red dots correspond to the electrode location, green and gray dots correspond to the reference and ground connections, respectively. On the left (**b**), a real picture of the EEG calp.

For EEG readings, an 8-channel semi-dry EEG cap (Versatile 8, BitBrain Technologies, Zaragoza, Spain) was used. The electrodes can be located anywhere on the international 10-10/10-20 system. For our study, the 8-channel configuration is shown in Figure 1a and described in Table 1. Figure 1b shows a picture of the cap with electrode connections. A detailed description of the EEG device can be found elsewhere [40].

### 2.3. EEG Processing

#### 2.3.1. Preprocessing

For each channel, the independent component analysis (ICA) algorithm [41] was applied to remove artifacts from the raw EEG signals sampled at 256 Hz. The EEG recordings were then normalized and filtered into the following five classical frequency bands: delta (0.5–4 Hz), theta (4–8 Hz), alpha (8–12 Hz), beta (12–30 Hz), and gamma (30–40 Hz).

A second-order Butterworth bandpass filter was applied to each frequency band using zero-phase filtering (filtfilt’ function in Matlab) to preserve phase characteristics. For each band, time- and frequency-domain features were calculated for each channel: power spectral density (PSD), average signal amplitude over time, excitability ratio (E/I ratio), and peak frequency.

#### 2.3.2. Average Band Power (ABP)

The average band power (ABP) is defined as the average power spectral density (PSD) across channels. PSD was calculated using a previously described methodology [40].

#### 2.3.3. Average EEG Signal Amplitude over Time

Spatial information from EEG signals is obtained by reconstructing EEG heatmaps, as described in reference [40], where each electrode location is assigned the average amplitude of the EEG signal for each channel over a 1 s integration time.

#### 2.3.4. Cortical Excitability Ratio

This work defines the cortical excitability ratio (CER) as the ratio of the computed ABP for the given visual stimuli to the previous visual condition (i.e., the control visual stimulus and the scattering free flicker chromatic/achromatic stimulation, respectively). Thus, for scattering-free stimulation (Equation (1)) CER is expressed as the ratio of the ABP for the chromatic/achromatic stimuli to the control value of the ABP for each wave band (f).

For scattering-induced visual stimulation, the cortical excitability ratio (CER_scatt_, Equation (2)) is computed as the ratio of the ABP for the chromatic/achromatic stimuli limited by scattering to their corresponding values of ABP (i.e., chromatic/achromatic stimuli) without scattering effects.

(1) $$CER\left(f_{i}\right)=\frac{{ABP}_{color}\left(f_{i}\right)}{{ABP}_{control}\left(f_{i}\right)}-1$$

(2) $${CER}_{scatt}\;\left(f_{i}\right)=\frac{{ABP}_{color+scatt}\left(f_{i}\right)}{{ABP}_{color}\left(f_{i}\right)}-1$$

If visual stimulation induces no relative cortical response, the CER will be zero. Positive and negative values are associated with cortical excitability and inhibitory response, respectively.

Let us suppose that CER_scatt_ and CER induce cortical excitability and inhibition for a given visual stimulus, respectively. We then propose a new way to measure the excitation/inhibition ratio (E/I) given by the following equation:

(3) $$\left(E/I\right)\;Ratio=\left(\frac{{CER}_{scatt}-CER}{{CER}_{scatt}+CER}\right)$$

#### 2.3.5. Peak Frequency Relative Shift

Peak frequency (f_peak_) is a spectral measure of EEG signals and represents the frequency at which the EEG signal exhibits the maximum power spectral density within a given waveband range. It is calculated as follows:

(4) $$f_{peak}={argmax}_{f}\left|FFT(f_{i})\right|$$

The frequency relative shift is defined as the displacement of the f_peak_ in a frequency band when visual conditions are altered.

### 2.4. Visual Stimulator

The visual device consists of a non-forming image Arduino-based flicker stimulator described in [40]. The device configuration allows for chromatic and achromatic stimulation at the visual critical flicker fusion frequency (CFF). As mentioned in the Introduction, the magnocellular visual pathway reaches its limit at this CFF frequency. M-pathway is inhibited for suprathreshold values, generating a compensatory response from the parvocellular system. Secondly, optical scattering was introduced as a natural degrader of spatial resolution and, therefore, an inhibitor of the parvocellular-pathway. Optical scattering was induced by Bangerter occlusion foils, commonly used for the treatment of amblyopia, which act as fog filters. As a consequence of forward scattering, the retinal image quality deteriorates depending on the level of transparency of the Bangerter foils [40].

### 2.5. Experimental Procedure

This study is a prospective exploratory experimental investigation. Control measurements consisted of EEG recordings, while the subject perceives a steady, punctual white visual stimulus. Each 10 s of the EEG recordings under different experimental conditions (see Table 2) were measured in one session for each subject. Subsequently, control EEG readings and those under different flickering visual stimuli from the same participants were compared to explore intra-subject variations.

**Figure 2:**
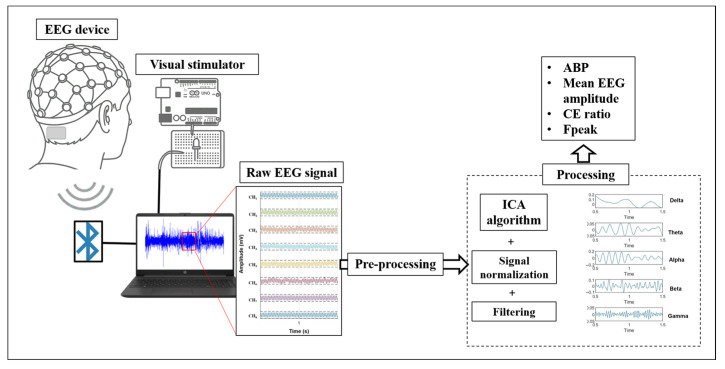
Schematic diagram of the experimental procedure.

Before EEG readings, the critical flicker fusion frequency was measured for each subject and experimental visual condition using the visual stimulator, following a previously described protocol [40]. Figure 2 outlines the experimental procedure. The experimental conditions are classified as shown in Table 2.

### 2.6. Data Analysis and Statistics

#### 2.6.1. One-Way Analysis of Variance (ANOVA)

One-way repeated measures analysis of variance (one-way RM ANOVA) was performed to obtain the F-statistic and *p*-value as indicators of statistical significance. While the F-value represents the ratio of variance between flicker stimuli and control values or between regular flicker stimulation and scattering induced flicker stimuli, the associated *p*-value indicates whether there is a significant difference in cortical activity between the repeated measures.

#### 2.6.2. Pearson Cross-Correlation Coefficient

Pearson’s cross-correlation coefficient was calculated to measure the correlation between two EEG signal temporal series (EEG (t)) using the Matlab function “corrcoef”. The outputs are the correlation coefficient (R) and the associated *p*-value, which assesses the statistical significance of the similarity.

#### 2.6.3. Root Mean Square Error (RMSE)

The RMSE quantifies the mean amplitude deviation between flicker-stimulated and control EEG signals (EEG_flicker_ and EEG_control_, respectively). A lower RMSE indicates greater morphological similarity in amplitude. N is the number of samples over the integrated time (t).

(5) $$RMSE=\sqrt{\frac{1}{N}\sum_{t=1}^{N}{\left({EEG}_{flicker}\left(t\right)\;-{\;EEG}_{control}(t)\right)}^{2}}$$

#### 2.6.4. Kolmogorov–Smirnov (K-S) Test

This non-parametric test was calculated to assess whether the two signals originate from the same distribution. The Matlab function “kstest2”was used.

Statistical analysis and graphical representation were performed using the Origin Lab scientific software 2024b (Origin Lab Corp., Northampton, MA, USA). EEG processing has been fully implemented in a custom-written Matlab script (Matlab 2019b, the MathWorks Inc., Natick, MA, USA).

## 3. Results

### 3.1. Critical Flicker Fusion Frequency Assessment

**Figure 3:**
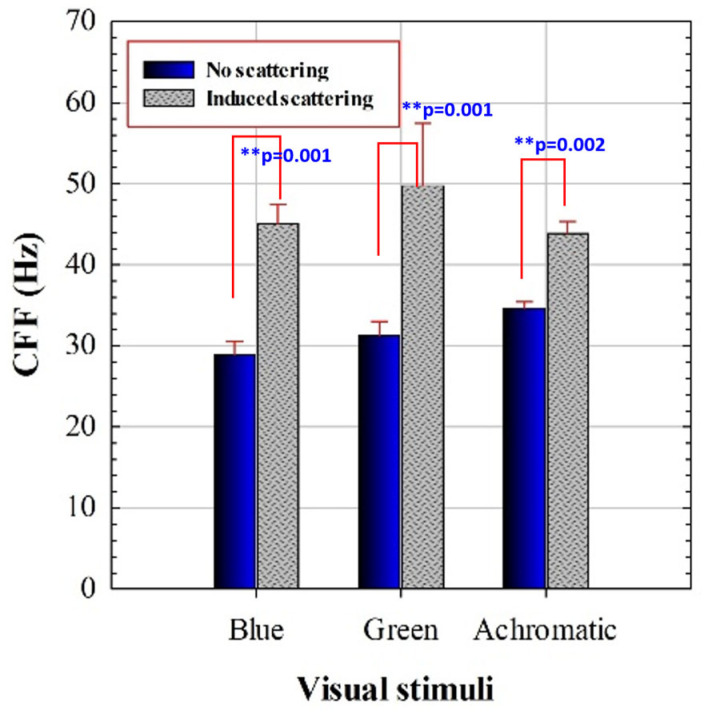
CFF for blue, green, and achromatic visual stimuli for regular and scatter-induced CFF assessment. Asterisks indicate statistical differences (significance level: ** *p* < 0.005). Error bars correspond to standard deviation of the mean CCF values.

Each subject presents a specific CFF threshold for each experimental condition. Figure 3 compares the mean CFF values with and without induced optical scattering for each presented stimulus. These new results reproduce the improvement of temporal resolution limit of vision due to scattering effects described in reference [40].

A 2 × 3 repeated measures ANOVA was conducted with stimulus (achromatic, blue, green) and scattering (no/yes) as within-subjects factors. The analysis revealed significant main effects of scattering (F(1,18) = 205.74, *p* < 0.0001), stimulus (F(2,36) = 9.19, *p* = 0.0018), and a significant stimulus × scattering interaction (F(2,36) = 10.51, *p* = 0.0009). Post hoc Bonferroni comparisons were conducted for the different chromatic conditions summarized in Table 3.

### 3.2. Averaged Band Power for Different Visual Stimuli

**Figure 4:**
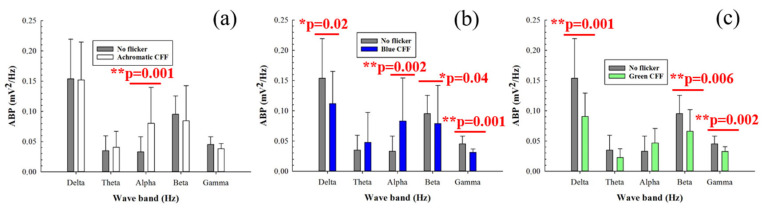
Average band power (ABP) as a function of the waveband for achromatic (**a**), blue (**b**), and green (**c**) CFF stimuli compared to the control steady visual state. Asterisks mark pairs for which a significant difference (*p* < 0.05) was found.

Figure 4 shows the average band power (ABP) calculated from the power spectrum density of the 8 electrodes as a function of the filtered band frequency for all participants. Specifically, it compares the control ABP values (gray bars) corresponding to a steady punctual visual stimulus with the ABP values for flicker stimuli at the critical flicker fusion frequency (CFF) for achromatic (Figure 4a), blue (Figure 4b), and green (Figure 4c) channels of the visual stimulator. Statistical analysis (one-way ANOVA test) revealed increased cortical alpha activity for the achromatic (Figure 4a) and blue flicker stimuli (Figure 4b). In contrast, flicker stimulation induced a reduction in neural activity for high-frequency bands (i.e., beta and gamma oscillations) for both blue and green flicker stimuli (Figure 4b,c, respectively). Furthermore, reduced delta activity was observed only for blue flicker light (Figure 4b). Regarding the influence of the stimulus on the ABP, the statistical analysis shown in Table 4 revealed that for all frequency bands with the exception of the beta range, the change between an achromatic stimulus and another achromatic (green) stimulus generates significant responses in the ABP.

**Figure 5:**
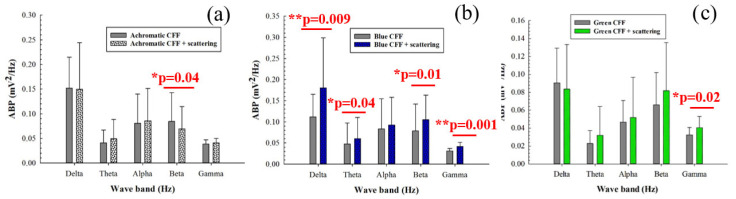
Average band power (ABP) as a function of the waveband for achromatic (**a**), blue (**b**), and green (**c**) CFF stimuli with scattering compared to scattering-free CFF stimuli. Asterisks mark pairs for which a significant difference (*p* < 0.05) was found.

When the parvocellular pathway of the visual function is inhibited by scattering degradation during visual stimulation, achromatic stimulation showed significant inhibition of beta activity (Figure 5a). Regarding chromatic stimuli, flicker blue light showed the opposite behavior compared to flicker stimulation without scattering (Figure 5b); a significant increase in cortical activity was observed in both low- (delta and theta bands) and high-frequency (beta and gamma oscillations) bands. The green flicker stimulus showed significant neural inhibition in the gamma band only (Figure 5c).

### 3.3. Cortical Excitability Ratio

**Figure 6:**
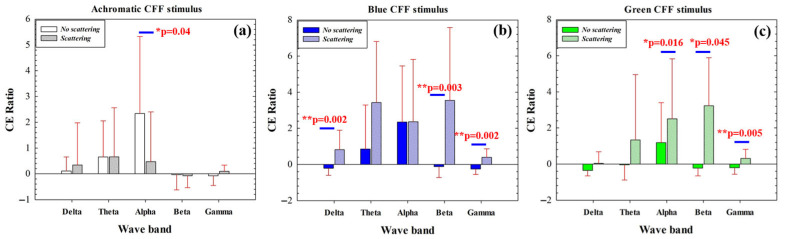
Comparison of CE ratio before and after applying optical scattering as a function of the waveband for achromatic (**a**), blue (**b**), and green (**c**) CFF stimuli. The asterisks mark where significant differences were found (significance level: * *p* < 0.05; ** *p* < 0.005).

The cortical excitability (CE) ratio measures the ability of our visual stimulator to induce cortical excitation or inhibition in a given waveband. Figure 6 compares the CE ratio before and after applying optical scattering for achromatic (Figure 6a), blue (Figure 6b), and green CFF (Figure 6c) visual stimulation. For the conditions without scattering, the CE parameter shows significant differences when alternating between the achromatic and green chromatic stimuli, as shown in Table 5.

For achromatic stimulation, maximal cortical excitation was observed for the alpha band while using scattering-free CFF stimulus; when scattering is introduced, CE is significantly reduced (Figure 6a). Chromatic stimulation showed an increase in CE as a consequence of optical scattering effects. The scattering-free flicker showed inhibition of the CE ratio for high-frequency bands (beta and gamma) for both blue (b) and green (c) stimuli. In contrast, the introduction of scattering revealed significant excitation of high-frequency neural oscillations (Figure 6b,c). This effect was also observed for the low-frequency delta band for the blue CFF stimulus only.

The data in Figure 6 show how the application and elimination of optical scattering as a function of the waveband for each stimulus condition in green and blue chromatic CFF visual stimulation allows modulation of high-frequency cortical activity. Specifically, the induction of optical scattering excites beta and gamma waves while its elimination inhibits this activity.

**Figure 7:**
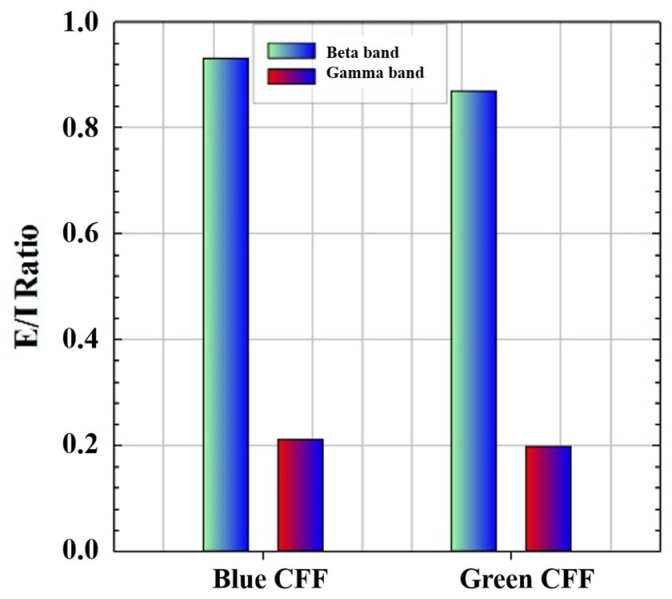
Proof of concept of E/I ratio calculation for high-frequency bands and two chromatic CFF visual stimuli.

Therefore, if we apply Equation (3) to the high-frequency CE values of the blue and green chromatic stimuli, we obtain, as proof of concept, the ability of the visual simulator to measure the excitation/inhibition (E/I) ratio as shown in Figure 7. Since the E/I parameter has been defined as the modulation contrast of excitability with respect to inhibition achieved through visual stimulation, the lower the E/I ratio, the lower the parameter value, and the more similar the response amplitudes of inhibition and excitation will be.

### 3.4. Topographic EEG Reconstruction

The previous sections showed the average of cortical activity of 8 electrodes measured by the ABP parameter for different experimental conditions (see Table 2). This section shows the average electrical activity measured at each electrode position reconstructed as a topographic image. For this purpose, a unique EEG file was obtained for each experimental condition, resulting from the average of all participants.

**Figure 8:**
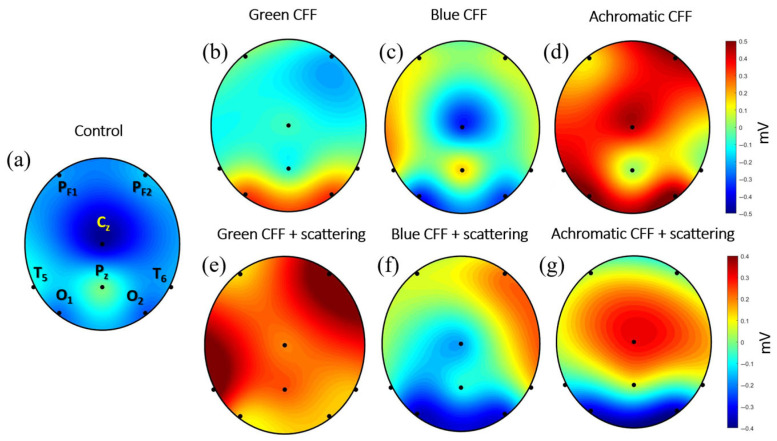
Topographic maps for the experimental visual conditions shown in Table 2. Black dots correspond to the electrode location. The scale bar is shown in millivolts.

The topographic maps shown in Figure 8 allow simultaneous visualization of brain activity modulation during visual stimulation in different cortical areas. It is noteworthy that EEG electrodes capture extracellular voltage. Excitatory neurotransmitters (glutamate) subsequently generate an excitatory postsynaptic potential that induces depolarization. In contrast, inhibitory neurotransmitters (GABA) generate inhibitory potentials that promote hyperpolarization. Therefore, depolarization and hyperpolarization of deep layers generate positive and negative EEG signals, respectively.

Figure 8 presents topographic maps of cortical electrical activity measured by EEG along the 8-electrode position described in Figure 1, reconstructed for different visual stimulation conditions. These maps provide a spatial perspective of how flickering visual stimulation in the CFF modulates brain activity under chromatic and achromatic stimuli, with and without parvocellular inhibition by induced light scattering. Figure 8a (control) shows a topography with a predominance of negative potentials; this pattern suggests neural cortical depolarization. This represents the average cortical baseline state in the absence of visual flicker stimulation.

Panels of Figure 8b–d show cortical responses to flickering stimuli in the CFF for green, blue, and achromatic visual stimuli, respectively. In (b) (green CFF), activity is concentrated in the occipital lobe, suggesting activation of visual areas sensitive to medium-wavelength stimuli. In (c) (blue CFF), a different distribution is observed, with a bilateral occipital activity and the sensorimotor cortex, likely reflecting contributions from the S-cone pathway. (d) (Achromatic CFF) shows the strongest and most widespread positive activity (red), especially in the occipital and parietal regions, indicating robust activation of the magnocellular pathway under luminance-based flicker.

Panels of Figure 8e–g show conditions under scattering-induced parvocellular inhibition. This manipulation is evident in the altered spatial distribution and magnitude of EEG responses. In (e) (green CFF + scattering), cortical activity increases dramatically in the right prefrontal and left temporal areas, shifting toward a widespread depolarization, as indicated by the strong positive values. This suggests either disinhibition or compensatory magnocellular activation. In (f) (blue CFF + scattering), a mixed pattern emerges, with both hyperpolarization in the visual cortex and depolarization in frontotemporal right area. (g) (achromatic CFF + scattering) maintains high positive amplitudes around central middle region occipital, but a strong hyperpolarization (inhibition) is observed in the occipital cortex.

### 3.5. Peak Frequency Relative Shift

**Figure 9:**
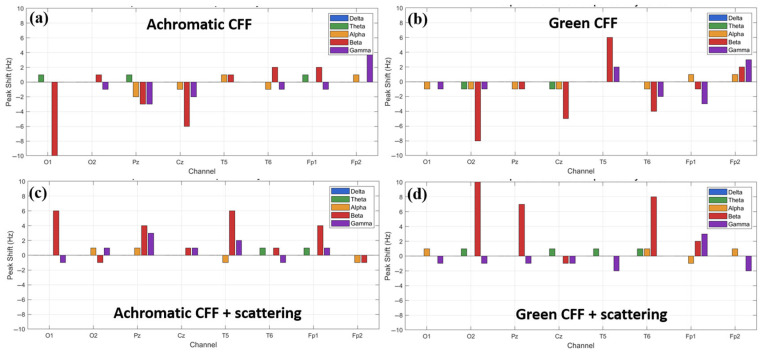
Relative shift peak frequency for all electrode locations as a function of the wave band and for different visual experimental conditions.

As observed above, different experimental conditions combining visual flicker stimulation with optical scattering have the ability to stimulate/inhibit cortical activity in different brain areas, mainly in high-frequency waves (i.e., beta and gamma oscillations). Therefore, it is logical to think that a shift in the predominant frequency peak for each specific waveband also occurs. Figure 9 shows the peak shift calculated for an achromatic and a chromatic (green) flicker stimulus in the CFF without (a, b) and with applied optical scattering (c, d) for each channel of the EEG cap as a function of the frequency band. For all conditions, the most representative maximum peak shift occurs in the beta and gamma bands. For the achromatic stimulus, the maximum shift is in the beta band and occurs in the visual cortex. The achromatic CFF stimulus without scattering (a) showed a negative shift, that is, towards the alpha band. However, when scattering is applied, the behavior is reversed, and the shift (although of smaller magnitude) occurs towards higher frequencies. Regarding the chromatic stimulus (green flickering light in the figure), the maximum peak shifts occur again in the beta band and are detected in the occipital lobe. The same behavior is observed as in the chromatic case: a negative shift in the case without scattering (b) and a positive shift when scattering is applied (d). Regarding the gamma oscillation, the maximum shift occurs in the prefrontal cortex, showing a similar trend to that of the beta band.

From the data shown in Figure 9, we might assume that a positive shift is associated with excitability and a negative shift with cortical inhibition. However, this is only true for beta waves; the opposite is true for gamma oscillations, as shown below.

**Figure 10:**
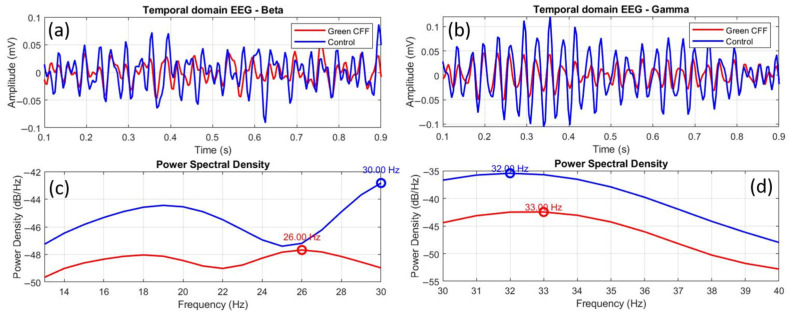
Comparative temporal and frequency domain EEGs between the control and green CFF visual conditions for beta (**a**,**c**) and gamma (**b**,**d**) wavebands. The overlapping circles on the power spectral density curves correspond to the dominant frequency peak, in each case.

Figure 10 shows comparative EEGs between control and scattering-free green flicker stimulation, in both the time and frequency domains, for the beta (a, c) and gamma (b, d) bands. The time-domain EEGs show a clear difference in amplitude between both conditions, but also a phase shift between oscillations in the case of beta waves (a). This phase shift is practically imperceptible in the gamma EEG (b), unlike the difference between the amplitudes. However, the frequency-domain EEGs show similar distributions in both cases but with a clear peak shift in the beta band (c) and a weak phase shift in the gamma range (d).

To quantify the graphical data in Figure 10, Table 6 shows the corresponding statistical and morphological analysis. Figure 9c,d show inhibited power density in beta and gamma waves for visual stimulation with green CFF with respect to the control state. The K-S test and the Pearson cross-correlation coefficient revealed statistical similarity but amplitude differences between the EEGs compared in Figure 9a,b.

Peak shifts of −4.0 and +1.0 Hz were observed for beta and gamma oscillations, respectively. While cortical inhibition in the beta range occurs with a negative shift in the frequency peak, this phenomenon is not observed in the gamma band, since, even with inhibition of oscillations due to the visual stimulation condition, the peak shift is displaced towards higher frequencies.

**Figure 11:**
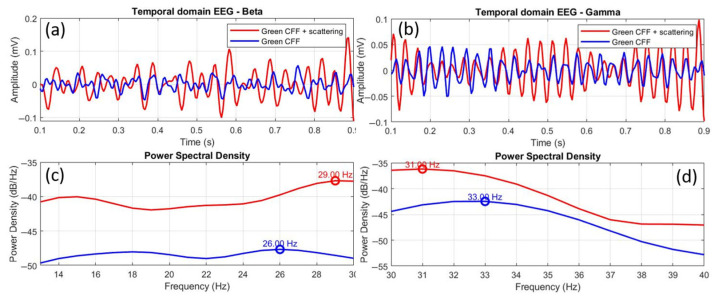
Comparative EEGs in temporal and frequency domains between the green CFF and scattering-induced green CFF visual conditions for beta (**a**,**c**) and gamma (**b**,**d**) wavebands. The circles superimposed on the power spectral density curves correspond to the dominant frequency peak, in each case.

Finally, Figure 11 shows the comparison of EEGs for the scattering-induced condition with respect to the scattering-free green CFF stimulation for beta (a, c) and gamma (b, d) waves. In this case, the time-domain EEGs show higher amplitude in both beta (a) and gamma (b) oscillations. Moreover, the power spectral density distributions showed more intense excitation for beta (c) than for gamma (d) bands. In the case of beta range, a positive peak shift of +3.0 Hz was found. Again, increased excitability is associated with a positive peak frequency shift. Furthermore, the Pearson cross-correlation coefficient, the K-S test, and the RMSE value showed that the EEG signals compared in Figure 10a differ statistically in morphological similarity and amplitude.

The scattering condition also induces excitability in the gamma range (Figure 11d), but, in a homologous manner to the previous case (stimulation with scatter-free green CFF), the frequency peak of the excited gamma oscillation with optical scattering is shifted in the opposite direction (−2.0 Hz) to the previous state of visual excitation (i.e., scattering-free green CFF). However, unlike in the beta range, the statistical tests (see Table 7) showed correlation between the EEGs shown in Figure 11b in similarity, but with differences in amplitude.

## 4. Discussion

Good retinal image quality is crucial for optimal spatiotemporal processing and color vision. The optical quality of the eye can be affected by three main factors: diffraction, optical aberrations, and scattering effects [42]. It is well-known how increased aberrations and scattering contributions degrade the spatial resolution [43], and it has been assumed for decades that these factors always systematically impair visual function as a whole.

However, our previous studies showed that both high-order aberrations and ocular scattering enhance the polychromatic temporal resolution of vision [24,40]. These antagonistic dynamics of the parvocellular and magnocellular pathways constitute the substrate and motivation of the present study.

In this work, we present empirical evidence that non-image-forming visual stimulation at the critical flicker fusion frequency (CFF), combined with optical scattering-induced spatial degradation, can modulate cortical excitability, particularly at high-frequency oscillations (i.e., beta and gamma).

We designed and employed an Arduino-controlled visual stimulator that delivers flickering achromatic, green, and blue stimuli in the CFF, with and without optical scattering spatial degradation, and subsequently measured cortical responses using EEG readings.

An initial statistical analysis revealed significant main effects of the stimulus (i.e., spectral range excitation), the scattering effects, and a significant stimulus-scattering interaction in measured critical flicker fusion frequency (see Figure 3).

Visual flicker stimulation has the ability to synchronize cortical neural oscillations via the thalamocortical pathway [44]. It is also well-known that neurons in the visual cortex react to flickering visual stimuli at the same frequency as the flickering light, producing the effect of neural oscillation or resonant frequencies [28]. This resonance phenomenon has been observed predominantly in the gamma frequency range and has the potential to improve cognitive function [45].

Adaikkan et al. [46] demonstrated that gamma entrainment with flicker visual stimulation offers neuroprotection in mouse models of neurodegeneration.

Our visual stimulation is delivered at critical flicker fusion frequency, close to the gamma band. Therefore, we are interested in focusing this discussion section on the effects of high-frequency bands.

In the absence of optical scattering, achromatic stimulation increased alpha band power, suggesting greater cortical activation at lower brain frequencies (Figure 4a). More interestingly, both blue and green CFF stimulation induced inhibition in higher frequency bands (beta and gamma, Figure 4b,c), suggesting that chromatic flicker stimulation selectively attenuates fast oscillatory brain activity.

In contrast, by introducing optical scattering (suppressing parvocellular input), the same chromatic flicker stimuli reverse their effects, significantly increasing beta and gamma activity (Figure 5b,c). This reversal suggests a compensatory recruitment of the magnocellular system under conditions of spatial degradation, consistent with the known high temporal sensitivity and low spatial acuity of the magnocellular pathway [15].

This dichotomy reinforces previous observations that the M- and P-pathways operate in antagonistic interaction [18], and our results show that manipulating spatial information through scattering can tilt this balance towards the temporal domain.

The cortical excitability ratio (CER) and the derived excitation/inhibition (E/I) ratio introduced in this study allow a quantifiable assessment of cortical dynamics in response to visual flicker stimulation. The modulation of high-frequency oscillations by scattering suggests a possible framework for exploring the neuroplastic mechanisms involved in rebalancing the E/I ratio, a critical factor in neurological and psychiatric disorders such as schizophrenia and autism spectrum [47].

EEG topographic maps further showed that optical scattering not only modifies spectral properties but also alters the spatial distribution of cortical activity, with notable engagement of occipital, prefrontal, and temporal regions under blue and green scattering-induced conditions. This suggests a broader network recruitment that could reflect compensatory responses or cross-modal plasticity, in line with models of cortical reorganization when one pathway is suppressed [48]. Our results are in line with the findings of Herrmann [28], who reported an increase in EEG response and glutamatergic receptor expression under high-frequency visual stimulation. Similarly, our peak-shift analysis revealed that beta inhibition is associated with negative frequency shifts (Figure 10), whereas beta excitation showed positive shifts (Figure 11), reinforcing the validity of peak frequency as an indicator of cortical excitability. In contrast, gamma oscillations demonstrate inverted behavior, showing amplitude changes but peak shifts in the opposite direction.

While most of the literature has focused on transcranial stimulation to modulate E/I balance [49], this study provides a low-cost contactless method that takes advantage of the intrinsic dynamic of the visual system. The role of the visual system in cognitive modulation is supported by studies linking magnocellular deficits to conditions like dyslexia [50], schizophrenia [51], and Alzheimer’s disease [52]. Our results indicate that targeted visual flicker with controlled scattering could represent a novel tool for investigating or even therapeutically modulating cortical networks affected in these conditions.

Several limitations must be acknowledged. First, the EEG system used, while practical and minimally invasive, offers limited spatial resolution and may not capture deeper cortical activity. Second, individual differences in CFF threshold and scattering perception introduce variability that may affect generalization.

Furthermore, while this work proposes the E/I ratio as a promising metric, its validation against other neurophysiological or behavioral markers remains pending.

## 5. Conclusions

This study presents evidence that flicker stimulation at the critical flicker fusion frequency, combined with optical scattering, modulates cortical excitability in a chromatic- and frequency-specific manner. Our results suggest that parvocellular suppression through spatial degradation induces compensatory magnocellular activation, reflected in increased beta and gamma oscillatory power.

We propose a novel, EEG-based E/I ratio metric that captures the dynamic balance of excitation and inhibition. This framework opens new directions for non-invasive neuromodulation techniques targeting visual and cognitive networks and offers translational potential for conditions characterized by altered cortical oscillations, such as Alzheimer’s disease, epilepsy, depression, and schizophrenia.

## Figures and Tables

**Table 1:** Electrode name, scalp location, and brief description of the corresponding brain area.

Electrode	Scalp Location	Brain Area	Description
#1: O1	Left occipital	Occipital lobe (left)	Visual processing
#2: O2	Right occipital	Occipital lobe (right)	Visual processing
#3: Pz	Midline parietal	Parietal lobe	Spatial attention, somatosensory integration, coordination
#4: Cz	Central midline	Sensorimotor cortex	Motor and somatosensory processing
#5: T5	Left temporal	Temporal lobe (left)	Auditory processing and language
#6: T6	Right temporal	Temporal lobe (right)	Auditory perception and nonverbal memory
#7: Fp1	Left prefrontal	Prefrontal cortex (left)	Attention, decision-making, emotional regulation
#8: Fp2	Right prefrontal	Prefrontal cortex (right)	Executive functions, emotional processing and inhibition

**Table 2:** Summary of the experimental conditions. Achromatic, blue and green CFF refer to the visual stimulus presented at the critical flicker fusion frequency (CFF) for the different chromatic configurations.

Condition	Chromatic/Achromatic	Optical Scattering
Control	Steady Achromatic	No
#1	Achromatic CFF	No
#2	Blue CFF	No
#3	Green CFF	No
#4	Achromatic CFF	Yes
#5	Blue CFF	Yes
#6	Green CFF	Yes

**Table 3:** Comparison (*t*-test, Bonferroni corrected for multiple comparisons) of the CFF conditions without scattering.

CFF Condition	Difference in Means	t-Statistics	*p*-Value	Significance
Achrom. vs. Blue	5.80	10.27	<0.001	Yes
Achrom. vs. Green	3.43	6.48	<0.001	Yes
Blue vs. Green	2.38	3.45	0.007	Yes

**Table 4:** Comparison of the ABP for the different conditions and wavebands without scattering (*t*-tests, Bonferroni corrected for multiple comparisons).

**Delta**	**Difference in Means**	**T**	***p***	**Significance**
Achrom. vs. Blue	0.04	2.16	0.045	Yes
Achrom. vs. Green	0.06	3.26	0.004	Yes
**Theta**	**Difference in Means**	**T**	***p***	**Significance**
Achrom. vs. Green	0.02	3.87	0.001	Yes
Blue vs. Green	0.03	2.11	0.049	Yes
**Alpha**	**Difference in Means**	**T**	***p***	**Significance**
Achrom. vs. Green	0.034	2.34	0.031	Yes
Blue vs. Green	0.04	2.35	0.030	Yes
**Gamma**	**Difference in Means**	**T**	***p***	**Significance**
Achrom. vs. Green	0.007	3.59	0.002	Yes

**Table 5:** Comparison of the CE ratio for the different conditions and wavebands without scattering (*t*-tests, Bonferroni corrected for multiple comparisons).

**Delta**	**Difference in Means**	**t-Statistics**	***p*-Value**	**Significance**
Achrom. vs. Blue	0.332	2.77	0.012	Yes
Ahcrom. vs. Green	0.465	3.69	0.001	Yes
**Theta**	**Difference in Means**	**t-Statistics**	***p*-Value**	**Significance**
Achrom. vs. Green	0.693	3.65	0.002	Yes
**Alpha**	**Difference in Means**	**t-Statistics**	***p*-Value**	**Significance**
Achrom. vs. Green	0.41	2.74	0.013	Yes
**Beta**	**Difference in Means**	**t-Statistics**	***p*-Value**	**Significance**
Achrom. vs. Green	0.195	2.20	0.040	Yes
**Gamma**	**Difference in Means**	**t-Statistics**	***p*-Value**	**Significance**
Achrom. vs. Green	0.181	3.67	0.001	Yes

**Table 6:** Peak shift, correlation coefficient (R), root mean square error (RMSE) and Kolmogorov–Smirnov (K-S) test corresponding to the comparison between control and green CFF signals shown in Figure 9.

	Peak Shift (Hz)	R	RMSE	K-S
Beta	−4.0	0.574, (*p* < 0.0001)	0.081	*p* = 0.00458
Gamma	+1.0	0.682, (*p* < 0.0001)	0.0587	*p* = 0.00001

**Table 7:** Peak shift, correlation coefficient (R), root mean square error (RMSE) and Kolmogorov–Smirnov (K-S) test corresponding to the comparison between control and green CFF signals shown in Figure 10.

	Peak Shift (Hz)	R	RMSE	K-S
Beta	+3.0	−0.0756, (*p* = 0.23)	0.1329	*p* = 0.00014
Gamma	−2.0	0.168, (*p* = 0.0077)	0.0774	*p* = 0.00458

## Data Availability

The original contributions presented in this study are included in the article. Further inquiries can be directed to the author.

## References

[1] Ly J., Gaggioni G., Chellappa S., Papachilleos S., Brzozowski A., Borsu C., Rosanova M., Sarasso S., Middleton B., Luxen A. (2016). Circadian regulation of human cortical excitability. Nat. Commun..

[2] Bridi M.C.D., Zong F.J., Min X., Luo N., Tran T., Qiu J., Severin D., Zhang X.-T., Wang G., Zhu Z.-J. (2020). Daily oscillation of the excitation-inhibition balance in visual cortical circuits. Neuron.

[3] Dinse H.R., Höffken O., Tegenthoff M. (2023). Cortical excitability in human somatosensory and visual cortex: Implications for plasticity and learning—A minireview. Front. Hum. Neurosci..

[4] Sohal V.S., Rubenstein J.L.R. (2019). Excitation-inhibition balance as a framework for investigating mechanisms in neuro-psychiatric disorders. Mol. Psychiatry.

[5] Uzunova G., Pallanti S., Hollander E. (2016). Excitatory/inhibitory imbalance in autism spectrum disorders: Implications for interventions and therapeutics. World J. Biol. Psychiatry.

[6] Goel A., Portera-Cailliau C. (2019). Autism in the balance: Elevated E-I ratio as a homeostatic stabilization of synaptic drive. Neuron.

[7] Gao R., Peterson E.J., Voytek B. (2017). Inferring synaptic excitation/inhibition balance from field potentials. NeuroImage.

[8] Maestú F., de Haan W., Busche M.A., deFelipe J. (2021). Neuronal excitation/inhibition imbalance: Core element of a translational perspective on Alzheimer pathophysiology. Ageing Res. Rev..

[9] Pellegrino G. (2024). Excitation/inhibition balance relates to cognitive function and gene expression in temporal lobe epilepsy: A high density EEG assessment with aperiodic exponent. Brain Commun..

[10] Li G., Hsu L.-M., Wu Y., Bozoki A.C., Shih Y.-Y.I., Yap P.-T. (2025). Revealing excitation-inhibition imbalance in Alzheimer’s disease using multiscale neural model inversion of resting-state functional MRI. Commun. Med..

[11] Sood A., Preeti K., Fernandes V., Khatri D.K., Singh S.B. (2021). Glia: A major player in glutamate-GABA dysregulation-mediated neurodegeneration. J. Neurosci. Res..

[12] Biermann L., Wunram H.L., Pokorny L., Breitinger E., Großheinrich N., Jarczok T.A., Bender S. (2022). Changes in the TMS-evoked potential N100 in the dorsolateral prefrontal cortex as a function of depression severity in adolescents. J. Neural Transm..

[13] Tao C., Zhang G.-W., Sun W.-J., Huang J.J., Zhang L.I., Tao H.W. (2024). Excitation-inhibition imbalance in medial preoptic area circuits underlies chronic stress-induced depression-like states. Nat. Commun..

[14] Howes O.D., Shatalina E. (2022). Integrating the neurodevelopmental and dopamine hypotheses of schizophrenia and the role of cortical excitation-inhibition balance. Biol. Psychiatry.

[15] Masri R.A., Grünert U., Martin P.R. (2020). Analysis of parvocellular and magnocellular visual pathways in human retina. J. Neurosci..

[16] Goodhew S.C., Boal H.L., Edwards M. (2014). A magnocellular contribution to conscious perception via temporal object segmentation. J. Exp. Psychol. Hum. Percept. Perform..

[17] Skottun B.C. (2015). On the use of spatial frequency to isolate contributions from the magnocellular and parvocellular systems and the dorsal and ventral cortical streams. Neurosci. Biobehav. Rev..

[18] Ichinose T., Habib S. (2022). ON and OFF signaling pathways in the retina and the visual system. Front. Ophthalmol..

[19] Yoonessi A., Yoonessi A. (2011). Functional assessment of magno, parvo and konio-cellular pathways; current state and future clinical applications. J. Ophthalmic Vis. Res..

[20] Teng M., Khoo A.L., Zhao Y.J., Abdin E., Mok Y.M., Lim B.P., Tor P.C. (2021). Neurostimulation therapies in major depressive disorder: A decision-analytic model. Early Interv. Psychiatry.

[21] Lundstrom B.N., Osman G.M., Starnes K., Gregg N.M., Simpson H.D. (2023). Emerging approaches in neurostimulation for epilepsy. Curr. Opin. Neurol..

[22] Monteiro F., Carvalho Ó., Sousa N., Silva F.S., Sotiropoulos I. (2022). Photobiomodulation and visual stimulation against cognitive decline and Alzheimer’s disease pathology: A systematic review. Alzheimer’s Dement. Transl. Res. Clin. Interv..

[23] Brunelin J., Adam O., Mondino M. (2022). Recent advances in noninvasive brain stimulation for schizophrenia. Curr. Opin. Psychiatry.

[24] Ávila F.J. (2024). An Arduino-powered device for the study of white perception beyond the visual chromatic critical flicker fusion frequency. J. Imaging.

[25] Mankowska N.D., Marcinkowska A.B., Waskow M., Sharma R.I., Kot J., Winklewski P.J. (2021). Critical flicker fusion frequency: A narrative review. Medicina.

[26] Peters J.L., Bavin E.L., Brown A., Crewther D.P., Crewther S.G. (2020). Flicker fusion thresholds as a clinical identifier of a magnocellular-deficit dyslexic subgroup. Sci. Rep..

[27] Ye J., Sinha P., Hou F., He X., Shen M., Lu F., Shao Y. (2021). Impact of temporal visual flicker on spatial contrast sensitivity in myopia. Front. Neurosci..

[28] Herrmann C.S. (2001). Human EEG responses to 1–100 Hz flicker: Resonance phenomena in visual cortex and their potential correlation to cognitive phenomena. Exp. Brain Res..

[29] Chen S., Lu H., Cheng C., Ye Z., Hua T. (2024). Rapidly repeated visual stimulation induces long-term potentiation of VEPs and increased content of membrane AMPA and NMDA receptors in the V1 cortex of cats. Front. Neurosci..

[30] Park Y., Lee K., Park J., Bin Bae J., Kim S.-S., Kim D.-W., Woo S.J., Yoo S., Kim K.W. (2022). Optimal flickering light stimulation for entraining gamma rhythms in older adults. Sci. Rep..

[31] Lee K., Park Y., Suh S.W., Kim S.-S., Kim D.-W., Lee J., Park J., Yoo S., Kim K.W. (2021). Optimal flickering light stimulation for entraining gamma waves in the human brain. Sci. Rep..

[32] Sahin L., Figueiro M.G. (2020). Flickering red-light stimulus for promoting coherent 40 Hz neural oscillation: A feasibility study. J. Alzheimer’s Dis..

[33] Blanco-Duque C., Chan D., Kahn M.C., Murdock M.H., Tsai L.H. (2024). Audiovisual gamma stimulation for the treatment of neurodegeneration. J. Intern. Med..

[34] Rodrigues-Amorim D., Bozzelli P.L., Kim T., Liu L., Gibson O., Yang C.-Y., Murdock M.H., Galiana-Melendez F., Schatz B., Davison A. (2024). Multisensory gamma stimulation mitigates the effects of demyelination induced by cuprizone in male mice. Nat. Commun..

[35] Zhao X., Huang L., Guo R., Liu Y., Zhao S., Guan S., Ge R., Cui S., Wang S., Wang J.-H. (2017). Coordinated plasticity among glutamatergic and GABAergic neurons and synapses in the barrel cortex is correlated to learning efficiency. Front. Cell. Neurosci..

[36] Schmitt L.I., Wimmer R.D., Nakajima M., Happ M., Mofakham S., Halassa M.M. (2017). Thalamic amplification of cortical connectivity sustains attentional control. Nature.

[37] Uhlhaas P.J., Singer W. (2006). Neural synchrony in brain disorders: Relevance for cognitive dysfunctions and pathophysiology. Neuron.

[38] Abhang P.A., Gawali B.W., Mehrotra S.C. (2016). Technical aspects of brain rhythms and speech parameters. Introduction to EEG- and Speech-Based Emotion Recognition.

[39] Sawatari A., Callaway E. (1996). Convergence of magno- and parvocellular pathways in layer 4B of macaque primary visual cortex. Nature.

[40] Ávila F.J. (2025). Scattering improves temporal resolution of vision: A pilot study on brain activity. Photonics.

[41] Iriarte J., Urrestarazu E., Valencia M., Alegre M., Malanda A., Viteri C., Artieda J. (2003). Independent component analysis as a tool to eliminate artifacts in EEG: A quantitative study. J. Clin. Neurophysiol..

[42] Navarro R. (2009). The optical design of the human eye: A critical review. J. Optom..

[43] Artal P. (2014). Optics of the eye and its impact in vision: A tutorial. Adv. Opt. Photonics.

[44] Adaikkan C., Tsai L.H. (2019). Gamma entrainment: Impact on neurocircuits, glia, and therapeutic opportunities. Trends Neurosci..

[45] Wang K., Chen K., Wei Z., Wang T., Wei A., Gao X., Qin Y., Zhu Y., Ge Y., Cui B. (2024). Visual light flicker stimulation: Enhancing alertness in sleep-deprived rats. Front. Neurosci..

[46] Adaikkan C., Middleton S.J., Marco A., Pao P.-C., Mathys H., Kim D.N.-W., Gao F., Young J.Z., Suk H.-J., Boyden E.S. (2019). Gamma entrainment binds higher-order brain regions and offers neuroprotection. Neuron.

[47] Gao R., Penzes P. (2015). Common mechanisms of excitatory and inhibitory imbalance in schizophrenia and autism spectrum disorders. Curr. Mol. Med..

[48] Mohammed H., Hollis E.R. (2018). Cortical reorganization of sensorimotor systems and the role of intracortical circuits after spinal cord injury. Neurotherapeutics.

[49] Lin S.N., Lien Y.R., Shibata K., Sasaki Y., Watanabe T., Lin C.-P., Chang L.-H. (2023). The phase of plasticity-induced neurochemical changes of high-frequency repetitive transcranial magnetic stimulation are different from visual perceptual learning. Sci. Rep..

[50] Stein J. (2001). The magnocellular theory of developmental dyslexia. Dyslexia.

[51] Martínez A., Hillyard S.A., Dias E.C., Hagler D.J., Butler P.D., Guilfoyle D.N., Jalbrzikowski M., Silipo G., Javitt D.C. (2008). Magnocellular pathway impairment in schizophrenia: Evidence from functional magnetic resonance imaging. J. Neurosci..

[52] Sartucci F., Borghetti D., Bocci T., Murri L., Orsini P., Porciatti V., Origlia N., Domenici L. (2010). Dysfunction of the magnocellular stream in Alzheimer’s disease evaluated by pattern electroretinograms and visual evoked potentials. Brain Res. Bull..

